# Incidental findings from cancer next generation sequencing panels

**DOI:** 10.1038/s41525-021-00224-6

**Published:** 2021-07-19

**Authors:** Nika Maani, Karen Panabaker, Jeanna M. McCuaig, Kathleen Buckley, Kara Semotiuk, Kirsten M. Farncombe, Peter Ainsworth, Seema Panchal, Bekim Sadikovic, Susan Randall Armel, Hanxin Lin, Raymond H. Kim

**Affiliations:** 1 Program for Genetics and Genome Biology, Hospital for Sick Children, Toronto, ON Canada; 2 Department of Molecular Genetics, University of Toronto, Toronto, ON Canada; 3 Medical Genetics Program of Southwestern Ontario, London Health Sciences Centre, London, ON Canada; 4 Familial Cancer Clinic, Princess Margaret Hospital Cancer Centre, Toronto, ON Canada; 5 Grand River Hospital, Kitchener, ON Canada; 6 Zane Cohen Centre for Digestive Diseases, Familial Gastrointestinal Cancer Registry, Mount Sinai Hospital, Toronto, ON Canada; 7 Toronto General Hospital Research Institute, University Health Network, Toronto, ON Canada; 8 Molecular Genetics Laboratory, Division of Molecular Diagnostics, London Health Sciences Centre, London, ON Canada; 9 Familial Breast Cancer Clinic, Mount Sinai Hospital, Toronto, ON Canada; 10 Department of Pathology and Laboratory Medicine, Western University, London, ON Canada; 11 Department of Medicine, University of Toronto, Toronto, ON Canada

**Keywords:** Cancer genetics, Genetic testing

## Abstract

Next-generation sequencing (NGS) technologies have facilitated multi-gene panel (MGP) testing to detect germline DNA variants in hereditary cancer patients. This sensitive technique can uncover unexpected, non-germline incidental findings indicative of mosaicism, clonal hematopoiesis (CH), or hematologic malignancies. A retrospective chart review was conducted to identify cases of incidental findings from NGS-MGP testing. Inclusion criteria included: 1) multiple pathogenic variants in the same patient; 2) pathogenic variants at a low allele fraction; and/or 3) the presence of pathogenic variants not consistent with family history. Secondary tissue analysis, complete blood count (CBC) and medical record review were conducted to further delineate the etiology of the pathogenic variants. Of 6060 NGS-MGP tests, 24 cases fulfilling our inclusion criteria were identified. Pathogenic variants were detected in *TP53, ATM, CHEK2, BRCA1* and *APC*. 18/24 (75.0%) patients were classified as CH, 3/24 (12.5%) as mosaic, 2/24 (8.3%) related to a hematologic malignancy, and 1/24 (4.2%) as true germline. We describe a case-specific workflow to identify and interpret the nature of incidental findings on NGS-MGP. This workflow will provide oncology and genetic clinics a practical guide for the management and counselling of patients with unexpected NGS-MGP findings.

## Introduction

Blood-based genetic testing is a mainstay in the approach of hereditary cancer assessment^1–7^. Such genetic testing typically involves the analysis of DNA isolated from peripheral blood leukocytes (PBLs)^3,8,9^, with the assumption that leukocyte DNA is representative of the germline^8–10^. Most hereditary cancer syndromes are inherited in a monoallelic fashion with an expected 50% allele fraction (AF)^8^. Compared to traditional Sanger sequencing, next generation sequencing (NGS) technologies and the development of multi-gene panel (MGP) testing have significantly improved assay sensitivity and enabled the ability to test multiple genes simultaneously^4,8,9,11^. In a minority of cases, atypical genetic test results can be observed following NGS-MGP testing, which are not consistent with a monoallelic germline variant. Examples include the detection of variants with low AFs (<30%)^3,8,9,12^, multiple pathogenic variants in the same individual, and genetic testing results which are not congruent with the reported family history^13,14^. When derived from the analysis of PBLs, these unexpected genetic results may indicate a *de novo* germline event^15^, or a genetic variant of non-germline origin. These results could be considered incidental findings^16^, as they are outside of the original purpose for which the test or procedure was conducted^17^. Non-germline findings include post-zygotic mosaicism, hematologic malignancy, or clonal hematopoesis (CH) (a newly recognized phenomenon where genetic changes accumulate in myeloid cells)^3,5,6,8^.

CH is a recently described phenomenon in which somatic variants accumulate in PBL due to aging and exposure to therapies such as chemotherapy and radiotherapy^8,18–20^. These somatic variants can lead to precursors for neoplasms of undetermined significance^18,21–23^. Clinically, a growing body of evidence has uncovered a link between CH and a two to four-fold increased risk of atherosclerotic disease^18,24–28^, along with an increased risk for the development of overt hematologic neoplasia and increased overall mortality in CH patients^29^. Mosaicism is typically defined as the presence of genetically distinct populations of cells within an individual, following acquired change(s) occurring shortly after zygote formation^30,31^. If these changes are present in the gametes and involve germline cells, they may be transmissible to offspring^9,10,30–32^. Postnatally, further somatic tissue-specific genetic changes can be acquired and can lead to the development of cancer^8–10^. When somatic variants are detected exclusively in PBLs, they can be indicative of a hematological malignancy or CH^8,9,33^ and may lead to clonal expansion in the blood^21,34^. PBL-isolated variants, however, are not transmissible to offspring and their identification is important as patients carrying such variants are managed differently from those harbouring germline variants.

Given the increased uptake of NGS-MGP testing, there exists a growing need for the development of a workflow to both identify and manage the clinical follow-up of patients who present with potentially non-germline incidental findings. To further explore the management of patients presenting with potential incidental findings following germline NGS-MGP testing, we performed a retrospective chart review of individuals who underwent hereditary cancer NGS-MGP testing in Ontario with findings suspicious of a non-germline incidental finding and propose a clinical workup.

## Results

### Patient population

Between January 1, 2016 and December 31, 2019, 6060 patients with a personal or strong family history of cancer who were referred to a hereditary cancer clinic in Ontario on suspicion of a hereditary cancer syndrome consented to have NGS-MGP testing. All 6060 patients underwent a germline comprehensive hereditary cancer NGS panel at the Molecular Genetics Laboratory at London Health Sciences Centre in Ontario, Canada. Of these 6060 patients, 24 (0.4%) fulfilled the inclusion criteria suggestive of an incidental finding (Table 1). For patients with unquantified low allele fractions from an external laboratory (Patients 2, 33, 40), NGS-MGP was repeated at London Health Sciences Centre. To better understand the origin of these variants, NGS-MGP was conducted on secondary tissues and blood collected at different time points. This secondary testing was done in conjunction with a review of the patient’s medical history (including complete blood counts [CBCs]) to monitor for the presence or potential development of a hematologic disorder.

**Table 1:** Cancer type, treatment, and family history of patients presenting with incidental findings identified through NGS-MGP testing on peripheral blood leukocyte (PBL)-derived DNA.

Patient ID	Sex	Ethnicity	Cancer	Age at Cancer Diagnosis	History of Systemic Chemotherapy (Y/N)	WBC (10^9^)	Family Cancer Hx
2	F	Ashkenazi Jewish	Breast	52	Y	6.8	FDR: 3 (Melanoma + Prostate; Basal Cell Carcinoma; Breast + Melanoma); SDR: 7 (Kidney; Prostate; Breast; Breast; Colon + Melanoma; Ovary; Breast)
			Breast	63			
3	F	English	Serous Ovarian	78	Y	4.9	FDR: 1 (Cervical)
5	F	English/Scottish/ German	Serous Ovarian	68	NA	NA	NA
6	F	British	None	NA	N	7.5	FDR: 1 (Ovarian); SDR: 1 (Breast)
7	F	Caucasian	Ductal Carcinoma In-Situ	37	N	6.2	SDR: 13 (Breast; Lung; Lung; Breast; Breast; Breast; Breast; Breast; Lung + Liver; Pancreas; Pancreas; Pancreas; Brain)
8	F	Irish/ English	Hodgkin’s Lymphoma	21 and 29	Y	7.5	SDR: 4 (Sarcoma; Lung; Prostate; Breast)
			Thyroid Cancer	40			
			Breast	65			
			Serous Ovarian	70			
10	F	Sephardic Jewish	Serous Ovarian	54	Y	12.2	FDR: 2 (Testicular; Prostate + Thyroid)
11	F	European, Ashkenazi Jewish	Breast	59	Y	84.9	FDR: 4 (Basal Cell Carcinoma; Bone; Basal Cell Carcinoma; Breast); SDR: 4 (PSU; Breast; Leukemia; Hodgkin’s Lymphoma)
			Chronic Lymphoblastic Leukemia	72			
12	F	Caucasian	IgA Kappa Myeloma	72	Y	5.6	FDR: 3 (Polyps; Polyps; Gastrointestinal) SDR: 5 (Leukemia; Lymphoma; Melanoma; Breast: Breast)
			Colon	79			
13	F	West Indies	Breast	51	Y	5.8	FDR: 1 (Breast + Lung); SDR: 5 (Breast; Ovarian; Lung; Lung; Ovarian)
14	F	Sri Lankan	Serous Ovarian	80	Y	NA	FDR: 1 (Stomach); SDR: 4 (Brain; Breast; Breast; Stomach)
15	F	Dutch	Serous Ovarian	70	NA	NA	None
16	F	East Indian	Ovarian	60	Y	4.9	SDR: 1 (Cervical)
18	F	Latvian, French, Ashkenazi Jewish	Serous Ovarian	78	Y	8.1	FDR: 1 (Small Cell); SDR: 2 (Bladder; Small Cell Bladder)
19	F	British/French	Colorectal Adenomas	62	N	10.6	FDR: 1 (Ovarian); SDR:1 (Colon)
22	F	Slovenian	Polycythemia Vera	52	Y	7.2	FDR 1: (Colon)
			Serous Uterine	70			
24	F	English/Irish	Breast	38	Y	8.0	FDR: 1 (Peritoneal); SDR: 7 (Breast, Breast; Breast; Breast; Breast+ Ovarian; Colon; Brain)
			Ovarian	54			
			Breast	63			
			Lung	71			
27	F	English/Scottish	Breast	48	Y	6.8	FDR: 2 (Breast; Gastric); SDR: 2 (Brain; Breast)
			Breast	56			
			Breast	67			
29	M	Eastern European	Prostate	53	Y	10.4	FDR: 2 (Breast; Melanoma); SDR: 3 (Leukemia; Leukemia + Prostate; Breast)
33	F	French Canadian	Breast	35	Y	4.1	FDR: 1 (Bladder); SDR: 6 (Bone; Lung; Lung + Liver; Skin; Breast; Lung)
			Serous Ovarian	56			
			Ovarian	58			
40	F	Caucasian	>100 Adenomas	29	N	5.3	FDR: 1 (Breast)
41	F	Scottish	Breast	77	Y	7.3	FDR: 2 (Lung; Lung); SDR: 5 (Breast; Breast; Unknown; Unknown; Unknown)
42	M	Caucasian	Breast (male)	72	Y	5.6	FDR: 1 (Skin); SDR: 5 (Skin; Skin; Pancreas; Brain; Brain)
43	F	Scottish/Irish	Breast	55	Y	10.7	FDR: 2 (Thyroid; Pancreas); SDR: 2 (Breast; Breast + Ovary)

*WBC* White Blood Cell Count, *Hx* History, *FDR* First Degree Relative, *SDR* Second Degree Relative, *Y/N* Yes/No, *NA* Not available.

### Genetic testing results

Twenty-four patients fulfilled the inclusion criteria and underwent further investigation of secondary tissues and an assessment for CH and leukemia (Table 2). Four out of twenty-four (16.6%) patients had multiple pathogenic variants (Category 1), 18/24 (75%) patients had low AFs (Category 2), and 17/24 (70%) patients whose family histories were not suggestive of Li-Fraumeni syndrome (non-Chompret) had *TP53* variants (Category 3). Of note, many patients fit into more than one category. Identified cases involved 5 different hereditary cancer genes; the most common being *TP53*. Of the 24 patients in our cohort, 17 (70%) had findings in *TP53*, 2 (8.3%) in *CHEK2*, 3 (12.5%) in *ATM*, 2 (8.3%) in *APC* and 2 (8.3%) in *BRCA1*. Two out of twenty-four (8.3%) patients had pathogenic variants in more than one gene. To understand the origin of these variants, peripheral blood was collected at different time points when possible, and multiple AFs were obtained. Heterozygous variants were expected to have an AF of approximately 50%; however, in the case of non-germline variants, the AF may significantly fluctuate.

**Table 2:** Summary of genetic findings, allele fractions, and clinical diagnoses in patients with incidental findings identified through NGS-MGP testing on peripheral blood leukocyte (PBL)-derived DNA.

Patient ID	Gene	PBL Variant		PBL Allele Fraction (Age)	Secondary Finding Category	Direct Skin Biopsy Allele Fraction	Cultured Fibroblast Allele Fraction	Tumour Tissue Allele Fraction	Dx Conclusion
2	*TP53*	c.524 G > A	p.(Arg175His)	5.8% (66) 3.8% (67) 1.3% (68)	2,3	0%	0%	NA	CH
3	*TP53*	c.747 G > T	p.(Arg249Ser)	13.9% (78)	2,3	0%	NA	0%	CH
				6.7% (79)					
5	*TP53*	c.742 C > T	p.(Arg248Trp)	12.0% (69)	2,3	NA	NA	NA	Likely CH (Deceased)
6	*TP53*	c.586 C > T	p.(Arg196*)	13.8% (56)	2,3	3.80%	NA	NA	Likely CH
				14.9% (56)					
7	*TP53*	c.743 G > A	p.(Arg248Gln)	23.4% (38)	2,3	NA	21%	NA	*TP53* mosaic
				21.5% (38)					
				25.2% (39)					
				21.2% (40)					
8	*TP53*	c.1118delA	p.(Lys373Argfs*49)	13.8% (71)	2,3	NA	NA	NA	Likely CH (Deceased)
10	*TP53*	c.733 G > A	p.(Gly245Ser)	32.7% (71)	2,3	*TP53*: c.880 G > T, p(Glu294*) 12.2%	All 3 *TP53* variants 0%	NA	CH
				32.8% (71)		*TP53*: c.380 C > T, p.(Ser127Phe) 6.2%			
11	*TP53*	c.659 A > G	p.(Tyr220Cys)	53.3% (74); 82.3% (75)	1,3	5.1%	0%	NA	Chronic Lymphocytic Leukemia-related
		c.(?_21-)_(*21_?)del whole gene deletion		35% (74); 40% (75)		NA (DNA poor quality)	0%		
12	*TP53*	c.818 G > A	p.(Arg273His)	27.4% (83)	2,3	2.90%	0%	NA	Multiple Myeloma related
13	*TP53*	c.542 G > A	p.(Arg181His)	10.6% (52)	2,3	2.20%	0%	NA	CH
				15.0% (53)					
14	*TP53*	c.537 T > A	p.(His179Gln)	17.8% (82)	2,3	NA	NA	NA	Likely CH (Deceased)
15	*TP53*	c.818 G > A	p.(Arg273His)	10.4% (70)	2,3	0% (muscle)	NA	NA	CH
16	*TP53*	c. 438 G > A	p.(Trp146*)	29.0% (63)	1,3	NA	NA	NA	Likely CH
		c.811 G > T	p.(Glu271*)	38.0% (63)					
18	*ATM*	c.(?_21)_(*21_?)del whole gene deletion		10-15% (80)	2	0%	0%	NA	CH
19	*ATM*	c.7736_7737insC	p.(Arg2579Serfs*7)	15.0% (62)	2	5%	NA	NA	Likely CH
22	*ATM*	c.(?_−21)_(*21_?)del whole gene deletion		20.0% (71)	2	NA	NA	NA	Likely CH (Deceased)
24	*CHEK2*	c.1111 C > T	p.(His371Tyr)	27.3% (69)	1,2	NA	NA	NA	Likely CH
	*BRCA1*	c.5503 C > T	p.(Arg1835*)	50% (69)					Likely germline *BRCA1*
27	*CHEK2*	c.684-1 G > A		11.7% (68)	2	0.87%	NA	NA	Likely CH
				10.2% (68)					
29	*TP53*	c. 375 G > A	p.T125T Splice	50% (53)	3	NA	50%	NA	Full germline
33	*BRCA1*	c.1195_1196delCA	p.(His339*)	23.6% (58)	2	27.0%	24%	NA	*BRCA1* mosaic
40	*APC*	c.1383_1390del insATGAATGA	p.(His462*)	7.2% (33)	2	NA	NA	present in duodenal polyp (no AF)	*APC* mosaic
41	*TP53*	c.743 G > A	p.(Arg248Gln)	47.5% (77)	1,3	NA	NA	3.5% (breast tumour)	Likely CH
	*APC*	c.(?_−20)_(*21_?)del whole gene deletion		~30% (77)				NA	
42	*TP53*	c.637 C > T	p.(Arg213*)	32.6% (73)	3	NA	0%	NA	CH
43	*TP53*	c.818 G > A	p.(Arg273His)	37.1% (75)	3	NA	0%	NA	CH

*PBL* Peripheral Blood Leukocyte, *Dx* Diagnosis, *CH* Clonal hematopoiesis.

### Secondary tissue analysis

Fibroblast culturing has been reported to boost allelic fractions of variants that confer a growth advantage, suggesting that fibroblasts harbouring pathogenic variants in tumour suppressor genes may be present in cultured samples^35^. To further delineate this in our cohort, patients underwent a skin biopsy (direct and/or cultured). Eleven out of twenty-four (45.8%) patients underwent NGS-MGP analysis of a direct skin punch biopsy. Of these, 7 (63.6%) had a paired cultured skin biopsy. Four out of eleven (36.3%) patients (11, 12, 13, and 18) harboured low levels of PBL variants in direct skin biopsy samples and upon fibroblast culturing, these variants were no longer observed (Table 2). This suggests PBL contamination of the direct skin punch biopsies and is suggestive of CH. This was also observed in Patient 10, in which an additional second and third pathogenic variant in *TP53* were identified in direct skin punch biopsy. These variants were different than those initially identified in PBL-derived DNA and in cultured fibroblasts (Table 2), suggesting the presence of sequencing artifacts or somatic events occurring in the skin. Sample mix-up was ruled out by the assessment of three unique SNPs on skin and blood samples and results were confirmed using an additional NGS chemistry and sequencer. Taken together, the two additional variants found in the direct skin biopsy were likely somatic events isolated to the skin which have been observed in healthy individuals^36–38^. Conversely, Patient 6 harboured low levels of a *TP53* variant in direct skin punch biopsy, suggestive of PBL contamination in the biopsy sample. A cultured fibroblast sample for this patient was not available and it was ultimately concluded that this patient likely had CH (Table 2).

Three out of twenty-four (12.5%) patients underwent tumour tissue analysis. Patient 40 was mosaic for *APC;* patient 41 had a *TP53* variant present in the tumour at a low level, but did not have the second *APC* variant (Table 2). This case was considered likely due to CH and the tumour results due to the presence of admixed leukocytes of CH origin in tumour tissues. This phenomenon has also been reported in other tumour sequencing studies^5,22^. Lastly, skin and tumour samples in Patient 3 were negative for the *TP53* variant detected in PBL, suggesting CH.

### Diagnostic algorithm and conclusions

**Fig. 1:**
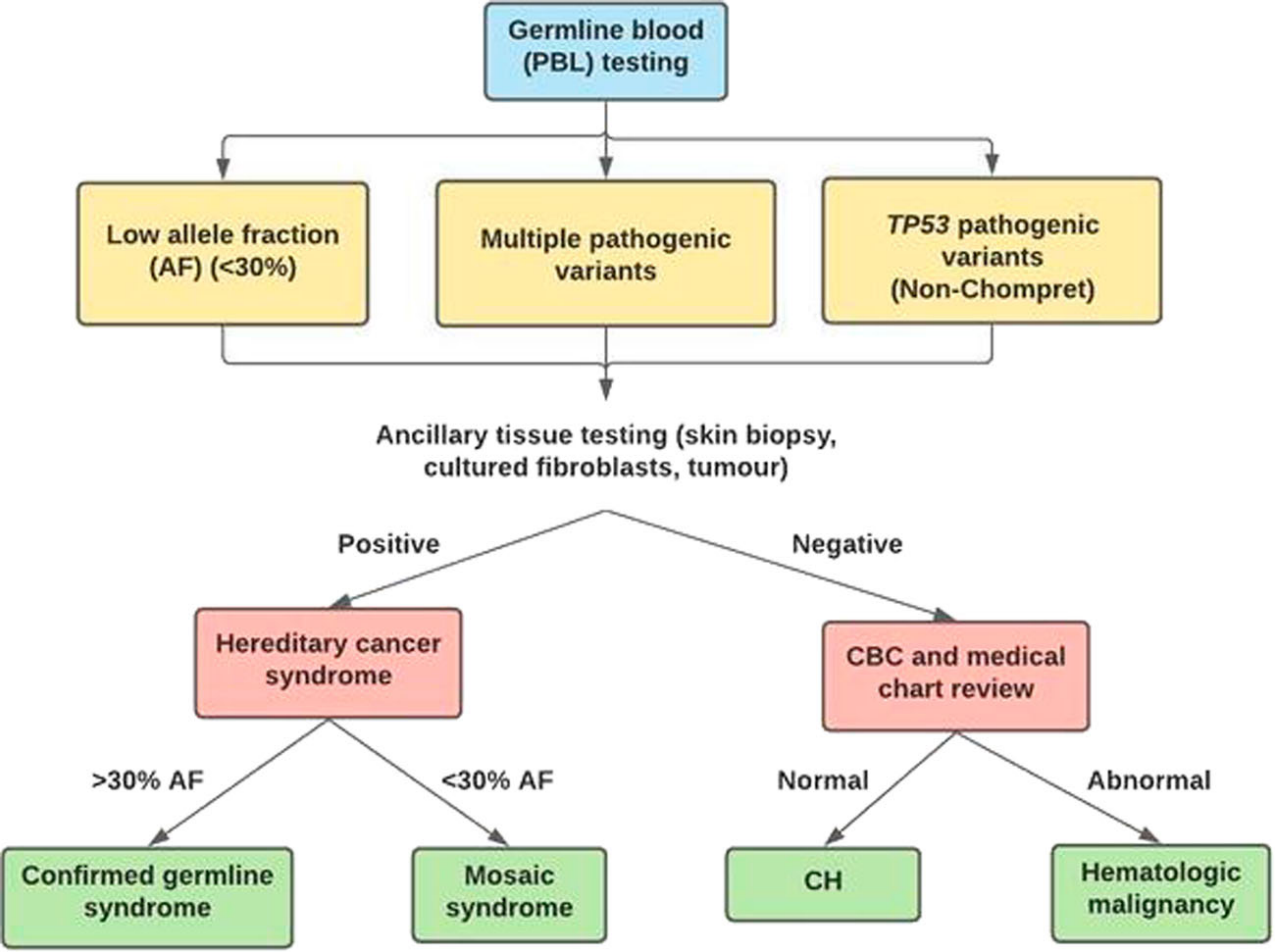
Genetic findings. Recommended diagnostic algorithm for patients presenting with potential incidental findings identified through NGS-MGP testing on peripheral blood leukocyte (PBL)-derived DNA. Genetic findings in individuals undergoing germline NGS-MGP testing on PBLs are classified in one of three categories and subsequently undergo further testing to identify downstream surveillance and diagnosis. This includes secondary tissue analysis (culture skin biopsy preferred) and medical chart review. CBC complete blood count.

Diagnostic conclusions for patients in our cohort were made using a specific algorithm (Fig. 1). Patients were considered to have a confirmed full germline hereditary cancer syndrome if the variant was found at >30% AF in PBL and cultured fibroblasts [1/24 (4.2%)]. Patients were classified as mosaic if the variant was detected at a < 30% AF in PBL-derived DNA and was also detected in a second tissue [3/24 (12.5%)]. Mosaic patients were clinically managed using relevant hereditary cancer syndrome screening guidelines.

Nine out of twenty-four (37.5%) patients had a variant with an AF ( < 30%) in PBL, which was either absent, or present in low levels in secondary tissues (results from cultured fibroblasts were used if there was a discrepancy with direct skin biopsy results). These patients were further evaluated for CH or a hematologic malignancy through a thorough review of CBC and medical charts (Fig. 1). If the CBC was normal and there was no evidence of hematologic malignancy upon medical chart review, patients were classified as CH [8/24 (33.3%)] (Fig. 1, Table 2). Molecular findings such as low AFs, normal CBC and exposure to chemotherapy were considered to support a diagnosis of CH. Fluctuating AF was also supportive of CH, however, this finding alone may be insufficient evidence of CH as the variance between timepoints was not assessed. Higher AFs and positive family history were considered suggestive of an inherited syndrome. Under this criteria, an additional 10 patients were classified as “likely CH” based on clinical history despite not having secondary tissue analysis. Combining these 10 patients with the 8 CH cases confirmed through secondary tissue analysis, 18/24 (75.0%) patients were concluded to have CH, representing a majority of our cohort. Of interest, 14/18 CH patients (77.7%) had a history of chemotherapy (Tables 1 and 2). In keeping with the ever-evolving clinical impact of CH, patients with findings suggestive of CH were enrolled into a research program assessing cardiovascular risk reduction^18,24–28^.

Lastly, 2 out of 24 (8.3%) patients were diagnosed with a hematologic malignancy. Patient 11 had an elevated white blood cell count and was diagnosed with chronic lymphoblastic leukemia whilst simultaneously undergoing germline testing. Patient 12 did not disclose their multiple myeloma diagnosis during the initial genetics consultation. Upon further review of their medical chart, it was noted they had been lost to follow-up regarding this diagnosis and was subsequently re-referred to their hematologist for ongoing care. These cases highlight the importance of a thorough medical history review for all patients undergoing a genetics assessment.

## Discussion

This retrospective chart review investigated incidental findings identified through NGS-MGP testing for hereditary cancer syndrome identification. These findings were considered incidental as they were unexpected from the original reason for NGS-MGP^17^. Genetic findings meriting further investigation for an incidental finding include: (1) multiple pathogenic variants in the same individual, (2) low AFs, and (3) pathogenic variants not consistent with personal and/or family cancer history (e.g. non-Chompret^8,39^ families harbouring *TP53* variants). We propose a clinical algorithm to determine the type of incidental finding and management of these cases (Fig. 1).

Aspects of our clinical workflow include secondary tissue analysis (cultured fibroblasts preferred) and a thorough review of CBC and medical records, builds on previously published algorithms^8^. The clinical implications of incidental findings can be significant and delineating their origin may directly impact patient management. Patients harbouring germline and mosaic pathogenic variants need to undergo annual radiological surveillance and/or surgical intervention. This is particularly relevant for *TP53* variants, which is the only gene on the NGS-MGP that is clearly associated with CH and overlaps with the highly penetrant hereditary Li-Fraumeni syndrome.

Based on their constellation of clinical findings, the majority of patients in our cohort were diagnosed as CH (33.3%) or likely CH (41.6%). Although CH is not considered a hematologic disorder, it represents a risk factor and a potential precursor for the evolution of various hematologic malignancies (myelodysplastic syndrome (MDS) and acute myeloid leukemia (AML))^40^, as well as cardiovascular complications^18,24–28^. Indeed, this emphasizes the importance of identifying and coordinating appropriate follow-up for CH patients, particularly those who have undergone chemotherapy. Of note, one CH patient in our cohort (Patient 6) was young (56 y) and had no history of chemotherapy or known cancer diagnosis at the time of testing (Table 1, Table 2). While rare, CH has been observed in healthy individuals <50 years of age^41^, demonstrating the importance of additional investigations for all patients with potential incidental findings, irrespective of age or cancer history.

In emphasizing certain cases, we seek to demonstrate complexities in the work-up of these patients, including some challenges in secondary tissue testing such as the limitations of direct skin punch biopsies and tumour tissue testing. These included, but were not limited to, the presence of somatic variants in the skin (Patient 10) and PBL contamination (Patients 12 and 13). Limitations of tumour testing included tissue infiltration of lymphocytes (Patient 41) and unreliable AFs (Patient 40). Indeed, streamlining secondary tissue testing is often complicated by logistical barriers in diagnostic laboratories. While many laboratories lack the appropriate resources to perform secondary tissue testing, the cases demonstrated here support the analysis of secondary tissues in certain clinical scenarios. This could include buccal swabs as an alternative to skin biopsy to distinguish CH from germline variants^42^.

The interpretation of incidental findings identified through NGS-MGP should be undertaken with high clinical suspicion, thorough genetic counselling and in communication with the molecular genetics laboratory and the patient. An example of this process is emphasized through a review of Patient 41. This case involved a family where genetic analysis of the proband identified an apparently heterozygous *TP53* gene mutation at an AF of 47.5%, a finding which was initially reported as a germline variant (Table 2). Upon genetic counsellor review, the family history did not fulfill Chompret’s Criteria^8,39^ and it became apparent that this result was not consistent with Li-Fraumeni Syndrome, likely representing an incidental finding. Through subsequent review of NGS-MGP data, an additional deletion in *APC* was identified at 30% allele fraction, strongly suggesting that the apparently heterozygous *TP53* variant was likely not germline. This was confirmed by the absence of the *TP53* variant in a secondary tissue (Table 2). Given that Patient 41 had no history of polyposis and was not initially counselled for the analysis of *APC*, this scenario emphasizes the importance of reviewing the patient’s cancer pedigree in context with genetic testing results. Genetic testing data that are secondary to the initial indication for testing may be of significant value in the interpretation of the final result for the patient. With the widespread use of NGS-MGP testing, analogous scenarios will become increasingly prevalent. Genetic counselling should involve a discussion of potential incidental results and thus raise awareness of the clinical implications.

Incidental findings represent a growing and unintended consequence of NGS-MGP testing in cancer patients. There exists a pressing need to properly advise patients of the different risks associated with incidental findings and the impact they may have on future care for themselves and their relatives. This includes patients with an atypical presentation of a germline hereditary cancer syndrome, post-zygotic mosaicism, CH, or hematologic malignancy. True germline and mosaic cases have implications for cancer risk in patients and their family members. In contrast, hematologic malignancies and CH require significantly different medical management strategies and are unlikely to affect family members. Moreover, with limited understanding of the etiology and significance of incidental findings, genetic testing of these variants should be offered to all offspring and other family members to determine transmission of the variant and assess segregation^13^.

Given that the use of NGS-MGP testing for cancer susceptibility genes has increased, the identification of incidental findings will become more pervasive amongst genetics clinics. To address this, we propose the adoption of a clinical workflow that will streamline the management of these patients. It is anticipated that future larger studies will demonstrate the impact of this workflow in wider populations.

## Methods

### Patient population

A retrospective chart review of patients who underwent NGS-MGP testing was conducted to identify genetic testing results suggestive of a non-germline incidental finding (mosaic, CH, hematologic malignancy). Patients with a personal and/or strong family history of hereditary cancer who underwent germline NGS-MGP testing at the clinicial diagnostic laboratory at London Health Sciences Centre between January 1, 2016 and December 31, 2019 were reviewed. The inclusion criteria were: Category 1) multiple pathogenic or likely pathogenic variants in the same individual; Category 2) presence of likely pathogenic or pathogenic variants at low AFs (10-30%); and/or Category 3) patients harbouring pathogenic *TP53* variants not fulfilling any of the criteria for LFS (i.e. Chompret criteria^8,39^). Of note, three patient samples (Patients 2, 33, 40) were initially tested at an external laboratory and NGS-MGP was repeated at London Health Sciences Centre. Participants not meeting the inclusion criteria were excluded. Ethical approval was obtained from all sites by the Research Ethics Committees (University Health Network REB: 18-5484; London Health Sciences Centre REB: R-20-354; Sinai Health System REB: 03-0231-U). A waiver of consent was provided by all three Research Ethics Committees.

### Data collection and analysis

For patients fulfilling an inclusion criteria, secondary tissues and/or blood collected at different time points were obtained when available for NGS-MGP testing. Secondary tissues included skin biopsy (with or without fibroblast culture), other normal tissues (e.g. muscle) and tumour tissue. Secondary tissue analysis was not conducted on patients who did not fulfill the inclusion criteria as their NGS-MGP results were not considered unexpected. A review of medical records was completed, including hematology, pathology and screening reports. Collected information included type of genetic testing, gene variant, date of referral for genetic testing, date of consultation, as well as cancer diagnoses, including modality of detection, pathology and treatment. Descriptive statistics were used to describe clinical findings in the patient population.

### Genetic testing

All genetic testing was done in a clinical molecular genetics laboratory (London Health Sciences Centre) and allele fractions were reported on all cases.

### DNA extraction

Genomic DNA from blood samples were isolated by standard protocols (MagNA Pure system, Roche Diagnostics, Laval, QC, Canada). DNA was quantified through absorbance with a DTX 880 Multimode Detector (Beckman Coulter, Brea, CA).

### Next-generation sequencing analysis

Genes included on the NGS-MPG panel include: *APC* (incl. 5’UTR), *ATM, BARD1, BMPR1A, BRCA1, BRCA2, BRIP1, CDH1, CDK4, CDKN2A, CHEK2, CTNNA1, EPCAM, FANCC, FANCM, FLCN, GREM1, HOXB13, MEN1, MLH1 (incl. 5’UTR), MSH2, MSH3, MSH6, MUTYH, NBN, NTHL1, PALB2, PMS2, POLE, POLD1, PTEN* (incl. 5’UTR)*, RAD51C, RAD51D, SDHB, SMAD4, STK11, TP53*. All coding exons and 20 base pairs of flanking non-coding sequences were enriched using a custom targeted hybrid protocol. Libraries were prepared with 100 ng of genomic DNA fragmented to 180 to 220 bp using a Covaris E220 Series Focused-ultrasonicator (Covaris, Inc., Woburn, MA). Each sample library was ligated with a specific barcode index according to the manufacturer’s protocol (Roche NimbleGen, Inc., Madison, WI) and assessed for quantification and size distribution using the Qubit fluorometer (Life Technologies) and 2200 TapeStation (Agilent Technologies, Santa Clara, CA) respectively. DNA libraries were pooled as 24-plex and captured using the SeqCap EZ Choice Library system (Roche NimbleGen, Inc.). Captured libraries underwent appropriate quality control analysis and were diluted to a concentration of 4nmol/L to process for sequencing according to the manufacturer’s instructions (Illumina, San Diego, CA). Library concentration for sequencing was 10pmol/L with a 1% PhiX spike-in. Libraries were sequenced using the MiSeq version 2 reagent kit, generating 2 x 150 bp paired-end reads using the MiSeq fastq generation mode (Illumina). The average coverage of the panel was between 500-1000x. For the direct skin biopsy for patient 10, to further assess the *TP53* variants found in the skin, a second NGS chemistry (Thermo-Fisher solid tumour Hotspot NGS panel) and second NGS sequencer (Ion Torrent S5) was used. Sequence alignment, variant calling and viewing were performed by the NextGENe and Geneticist Assistant software (Softgenetics). Variant interpretation and classification followed ACMG guidelines. Allele fractions of 30% were considered the empirical low threshold for heterozygous calling. This was established based on validation conducted on single nucleotide variants from NGS-MGP panels. While less reliable for copy number variants, a similar allele fraction threshold was used for exon-level deletions and MLPA was used to confirm (below). NGS allele fractions of 30%-70% were considered heterozygous.

### Multiplex ligation dependent probe amplification (MLPA) Analysis

For patients with exon-level deletions on NGS (11, 18, 22, 41) MPLA was used to confirm these findings. Genomic DNA (100 ng) was amplified according to the manufacturer’s recommendations (SALSA MLPA kit, MRC Holland, Amsterdam, the Netherlands). PCR products were separated by capillary electrophoresis (ABI 3730, Life Technologies, Thermo Fisher Scientific, Waltham, MA). Copy number alterations were analyzed with Coffalyser. Net software version 131211.1524 (MRC Holland).

### Sanger sequencing

Sanger sequencing was used for all cases to confirm the SNVs or small indels identified by NGS panel with >5% of allele fractions. Coding regions and flanking intronic regions (−20bp to +10 bp) were PCR-amplified and sequenced (BigDye Terminator v1.1 cycle sequencing kit, Life Technologies). Sequencing products were separated by capillary electrophoresis on an ABI3730 (Life Technologies) and analyzed with Mutation Surveyor v4.0.7 software (SoftGenetics).

### Reporting summary

Further information on research design is available in the Nature Research Reporting Summary linked to this article.

## Supplementary information

Reporting Summary

## Data Availability

The data that support the findings of this study are available on request from the corresponding author. Raw next-generation sequencing data has been deposited in Sequence Read Archive (SRA): SUB9747032, BioProject #PRJNA734018.
