# Supplementary material for: Incidental findings from cancer next generation sequencing panels
Source: NPJ Genom Med. 2021 Jul 19;6:63. doi: 10.1038/s41525-021-00224-6 (PMC8289933; doi:10.1038/s41525-021-00224-6)
Supplement: Supplementary file 1 — Reporting Summary [file 41525_2021_224_MOESM1_ESM.pdf]

nature research

Corresponding author(s): Dr. Raymond H. Kim and Dr. Hanxin Lin

Last updated by author(s): Apr 5, 2021

Reporting Summary

Nature Research wishes to improve the reproducibility of the work that we publish. This form provides structure for consistency and transparency in reporting. For further information on Nature Research policies, see our Editorial Policies and the Editorial Policy Checklist.

Statistics

For all statistical analyses, confirm that the following items are present in the figure legend, table legend, main text, or Methods section.

n/a	Confirmed	
☒	☐	The exact sample size (*n*) for each experimental group/condition, given as a discrete number and unit of measurement
☐	☐	A statement on whether measurements were taken from distinct samples or whether the same sample was measured repeatedly
☒	☐	The statistical test(s) used AND whether they are one- or two-sided<br>*Only common tests should be described solely by name; describe more complex techniques in the Methods section.*
☒	☐	A description of all covariates tested
☒	☐	A description of any assumptions or corrections, such as tests of normality and adjustment for multiple comparisons
☒	☐	A full description of the statistical parameters including central tendency (e.g. means) or other basic estimates (e.g. regression coefficient) AND variation (e.g. standard deviation) or associated estimates of uncertainty (e.g. confidence intervals)
☒	☐	For null hypothesis testing, the test statistic (e.g. *F*, *t*, *r*) with confidence intervals, effect sizes, degrees of freedom and *P* value noted<br>*Give P values as exact values whenever suitable.*
☒	☐	For Bayesian analysis, information on the choice of priors and Markov chain Monte Carlo settings
☒	☐	For hierarchical and complex designs, identification of the appropriate level for tests and full reporting of outcomes
☒	☐	Estimates of effect sizes (e.g. Cohen's *d*, Pearson's *r*), indicating how they were calculated

Our web collection on statistics for biologists contains articles on many of the points above.

Software and code

Policy information about availability of computer code

Data collection	A search of electronic patient records (EPR) at both the University Health Network (UHN) and the London Health Sciences Centre (LHSC) was conducted to identify individuals who met eligibility criteria. EPR access was granted exclusively though institutional VPN.
Data analysis	Patient Data: Descriptive statistics were used to outline clinical findings in the patient population. Sequencing Data: DNA libraries were pooled as 24-plex and captured using the SeqCap EZ Choice Library system (Roche NimbleGen, Inc.); sequence alignment, variant calling and viewing were performed by the NextGENe and Geneticist Assistant software (Softgenetics); copy number alterations were analyzed with Coffalyser. Net software version 131211.1524 (MRC Holland); sequencing products were analyzed with Mutation Surveyor v4.0.7 software (SoftGenetics).

For manuscripts utilizing custom algorithms or software that are central to the research but not yet described in published literature, software must be made available to editors and reviewers. We strongly encourage code deposition in a community repository (e.g. GitHub). See the Nature Research guidelines for submitting code & software for further information.

Data

Policy information about availability of data

All manuscripts must include a data availability statement. This statement should provide the following information, where applicable:

- Accession codes, unique identifiers, or web links for publicly available datasets
- A list of figures that have associated raw data
- A description of any restrictions on data availability

The data that support the findings of this study are available on request from the corresponding author. The data are not publicly available due to privacy or ethical restrictions.

Field-specific reporting

Please select the one below that is the best fit for your research. If you are not sure, read the appropriate sections before making your selection.

☒ Life sciences ☐ Behavioural & social sciences ☐ Ecological, evolutionary & environmental sciences

For a reference copy of the document with all sections, see nature.com/documents/nr-reporting-summary-flat.pdf

Life sciences study design

All studies must disclose on these points even when the disclosure is negative.

Sample size	A retrospective chart review was conducted on 6060 genetic testing samples which underwent clinical genetic testing at the study's clinical genetics laboratory. 24 cases fulfilled the inclusion criteria, therefore were included in the study.
Data exclusions	Inclusion criteria were the following: Category 1) multiple pathogenic or likely pathogenic variants in the same individual; Category 2) presence of likely pathogenic or pathogenic variants at low AFs (10-30%); and/or Category 3) patients harbouring pathogenic TP53 variants not fulfilling any of the criteria for LFS (i.e. Chompret criteria). Participants not meeting the inclusion criteria were excluded.
Replication	Reproducibility of findings was not relevant to this study given that this study was exclusively descriptive and not experimental.
Randomization	Randomization of the participants was not relevant to this study given that this study was exclusively descriptive and not experimental.
Blinding	Patient names were removed and patients were anonymized for study investigators responsible for compiling and describing the dataset.

Reporting for specific materials, systems and methods

We require information from authors about some types of materials, experimental systems and methods used in many studies. Here, indicate whether each material, system or method listed is relevant to your study. If you are not sure if a list item applies to your research, read the appropriate section before selecting a response.

Materials & experimental systems

n/a	Involved in the study	
☒	☐	Antibodies
☒	☐	Eukaryotic cell lines
☒	☐	Palaeontology and archaeology
☒	☐	Animals and other organisms
☐	☒	Human research participants
☒	☐	Clinical data
☒	☐	Dual use research of concern

Methods

n/a	Involved in the study	
☒	☐	ChIP-seq
☒	☐	Flow cytometry
☒	☐	MRI-based neuroimaging

Human research participants

Policy information about studies involving human research participants

Population characteristics	The population were patients who underwent germline genetic testing at the study's clinical genetics laboratory over the period of the study, January 1, 2016-Dec 31, 2019.
Recruitment	Inclusion criteria were delineated, and patients fulfilling the criteria underwent further genetic investigations as part of standard of care in the cancer genetics clinics.
Ethics oversight	Ethical approval was obtained from all sites by the Research Ethics Committees (University Health Network REB: 18-5484; London Health Sciences Centre REB: R-20-354; Sinai Health System REB: 03-0231-U).

Note that full information on the approval of the study protocol must also be provided in the manuscript.
